# Inositol monophosphate phosphatase genes of *Mycobacterium tuberculosis*

**DOI:** 10.1186/1471-2180-10-50

**Published:** 2010-02-18

**Authors:** Farahnaz Movahedzadeh, Paul R Wheeler, Premkumar Dinadayala, Yossef Av-Gay, Tanya Parish, Mamadou Daffé, Neil G Stoker

**Affiliations:** 1 Department of Pathology and Infectious Diseases, Royal Veterinary College, Royal College Street, London NW1 0TU, UK; 2 Tuberculosis Research, Veterinary Laboratories Agency, New Haw, Addlestone, Surrey KT15 3NB, UK; 3 CNRS, Institut de Pharmacologie et Biologie Structurale, UMR CNRS-Université Paul Sabatier (UMR 5089), 205, route de Narbonne, 31077 Toulouse cedex 04, France; 4 Department of Medicine, Division of Infectious Diseases, University of British Columbia, 2733 Heather St., Vancouver, British Columbia, Canada V5Z 3J5; 5 Queen Mary University of London, Barts and the London School of Medicine and Dentistry, 4 Newark Street, London E1 2AT, UK; 6 Current address: Institute for Tuberculosis Research (M/C 964), College of Pharmacy, Rm 412, University of Illinois at Chicago, 833 S. Wood St. Chicago, Illinois USA 60612-7231

## Abstract

**Background:**

Mycobacteria use inositol in phosphatidylinositol, for anchoring lipoarabinomannan (LAM), lipomannan (LM) and phosphatidylinosotol mannosides (PIMs) in the cell envelope, and for the production of mycothiol, which maintains the redox balance of the cell. Inositol is synthesized by conversion of glucose-6-phosphate to inositol-1-phosphate, followed by dephosphorylation by inositol monophosphate phosphatases (IMPases) to form *myo*-inositol. To gain insight into how *Mycobacterium tuberculosis *synthesises inositol we carried out genetic analysis of the four IMPase homologues that are present in the *Mycobacterium tuberculosis *genome.

**Results:**

Mutants lacking either *impA *(*Rv1604*) or *suhB *(*Rv2701c*) were isolated in the absence of exogenous inositol, and no differences in levels of PIMs, LM, LAM or mycothiol were observed. Mutagenesis of *cysQ (Rv2131c) *was initially unsuccessful, but was possible when a porin-like gene of *Mycobacterium smegmatis *was expressed, and also by gene switching in the merodiploid strain. In contrast, we could only obtain mutations in *impC *(*Rv3137*) when a second functional copy was provided in *trans*, even when exogenous inositol was provided. Experiments to obtain a mutant in the presence of a second copy of *impC *containing an active-site mutation, in the presence of porin-like gene of *M. smegmatis*, or in the absence of inositol 1-phosphate synthase activity, were also unsuccessful. We showed that all four genes are expressed, although at different levels, and levels of inositol phosphatase activity did not fall significantly in any of the mutants obtained.

**Conclusions:**

We have shown that neither *impA, suhB *nor *cysQ *is solely responsible for inositol synthesis. In contrast, we show that *impC *is essential for mycobacterial growth under the conditions we used, and suggest it may be required in the early stages of mycothiol synthesis.

## Background

*Mycobacterium tuberculosis *is a major global pathogen. In 2007, approximately 1.7 million deaths were caused by tuberculosis (TB) and an estimated 9.3 million people acquired the infection [1]. Patients can usually be cured through a six month course of a multiple drug regimen [2]. The efficacy of chemotherapy has however been compromised by the appearance of multi- and extensively drug resistant strains [3,4]. The search for potential novel drug targets and the subsequent development of new antibiotics is therefore urgent. Ideal candidates would be mycobacterial-specific and include pathways involved in the biosynthesis of the unusual cell envelope [5,6]; the target of some existing antibiotics, including isoniazid, ethionamide, ethambutol and pyrazinamide [7].

Inositol is a polyol that is not synthesized in most bacterial species. However, in the mycobacteria, inositol is found in lipoarabinomannan (LAM), a lipoglycan that is present in high levels in the cell envelope. LAM is composed of a mannan backbone with branched arabinosyl chains. It is anchored in the cell envelope by means of a phosphatidylinositol (PI) moiety. Other lipoglycans found in the cell envelope include lipomannan (LM) and PI mannosides (PIMs). PI-containing molecules have been demonstrated as essential for growth in the fast-growing species *Mycobacterium smegmatis*, as mutants lacking PI synthase are not viable [8].

The function of LAM in cell envelope integrity is unknown, but evidence suggests that it has profound effects on the host., for example, it stimulates macrophages to produce TNFα [9], nitric oxide [10], and matrix metalloproteinases [11]. LAM may therefore play a major role in the stimulation of an inappropriate host immune response, leading to the pathology that is characteristic of TB. LAM also induces transcriptional activation of HIV-1 [12,13] and may play a role in the synergy seen between HIV and TB. In addition to these effects, LAM is a major antigen [14,15]. While some PIMs are probable precursors of LAM, they may also have important functions of their own. PI dimannoside (PIM_2_), for example, has been implicated as a receptor for interacting with mammalian cells [16], as a secreted activator of Toll-like receptor 2 in macrophages leading to TNFα induction [17], and as an inducer of granuloma formation [18].

Inositol is also a constituent of the major mycobacterial thiol, mycothiol (1-D-*myo*-inosityl-2- [*N*-acetyl-L-cysteinyl] amido-2-deoxy-α-D-glucopyranoside) [19,20], which helps maintain the redox state of the cell and detoxifies harmful molecules. A mutant of *M. smegmatis *that essentially fails to produce mycothiol is viable, but grows poorly, and is sensitive to H_2_O_2 _[20] However, in *M. tuberculosis *the *mshA *and *mshC *genes, required for mycothiol biosynthesis, are essential genes [21,22]. Mycothiol may be more important in pathogenic mycobacteria as during infection they would be exposed to reactive oxygen intermediates within the macrophage.

The biosynthesis of inositol normally occurs in two steps. In the first, glucose-6-phospate is converted to inositol-1-phosphate (I-1-P) by inositol phosphate synthase (*Ino1*). We have shown previously that an *ino1 *(*Rv0046c*) mutant of *M. tuberculosis *is an inositol auxotroph, and is severely attenuated *in vivo *[23]. In the second step, the I-1-P is dephosphorylated by an inositol monophosphate phosphatase (IMPase) to form inositol. Previously, we identified the *M. smegmatis impA *gene, which is predicted to encode an IMPase, and showed that inactivation of this gene resulted in an altered colony morphology, reduced levels of PI dimannoside (PIM_2_), and altered permeability of the cell wall. This data suggests that *impA *is partly responsible for inositol synthesis in this species, presumably compensated by the presence of other *imp *genes [24]. In this paper, we describe the genetic analysis of four IMPase homologues *of M. tuberculosis*. We demonstrate that three, *impA*, *suhB *and *cysQ *are dispensible, while *impC *is essential, even in the presence of exogenous inositol.

## Methods

### Bacterial strains, plasmids and media

Bacterial strains and plasmids used are shown in Table 1. *M. tuberculosis *H37Rv (ATCC 25618) was cultured on Middlebrook 7H10 agar plus 10% (vol/vol) oleic acid-albumin-dextrose-catalse (OADC) supplement (Becton Dickinson). Middlebrook 7H9 broth (Difco) plus 10% (vol/vol) OADC supplement and 0.05% (wt/vol) Tween 80 was used to grow liquid cultures. Hygromycin (100 μg ml^-1^), kanamycin (20 μg ml^-1^), gentamicin (10 μg ml^-1^) and X-Gal (5-bromo-4-chloro-3-indolyl-β-D-galactopyranoside) at 50 μg ml^-1^, were added where appropriate. For supplementation with inositol, a 14% stock (w/v) (0.77 M) of *myo*-inositol (Sigma) was prepared and filter-sterilised. *E. coli *DH5α was used for all plasmid constructions.

**Table 1:** *M. tuberculosis *strains and plasmids

Strains/plasmids	Characteristics	Source
*E. coli *DH5α		Invitrogen

*M. tuberculosis *H37Rv	wild-type laboratory strain	ATCC 25618

FAME1	*M. tuberculosis suhBΔ*	This study

FAME2	*M. tuberculosis impAΔ*	This study

FAME4	*M. tuberculosis impCΔ*::pFM96	This study

FAME7	*M. tuberculosis*::pFM54 (*impCΔ *SCO)	This study

FAME9	FAME7 ::pFM96	This study

FAME11	FAME7::pFM123	This study

FAME63	FAME7::FM203	This study

FAME5	*M. tuberculosis ino1Δ*	[23]

FAME12	*M. tuberculosis ino1Δ*::pFM54 (SCO)	This study

FAME35	*M. tuberculosis*::pFM151 (*cysQΔ *SCO)	This study

FAME43	FAME35::FM164	This study

FAME53	*cysQΔ*::FM164	This study

FAME87	FAME35::FM203	This study

FAME93	*cysQΔ*::FM203	This study

FAME 120	*M. tuberculosis* *cysQΔ*:: pUC-Hyg-int	This study

pBluescript II SK+		Stratagene

pGEM5		Promega

pUC-Gm-int	pUC-based plasmid with *Hin*dIII cassette carrying *gm *and L5 *int*	[54]

pUC-Hyg-int	pUC-based plasmid with *Hin*dIII cassette carrying *hyg *and L5 *int*	[54]

p2NIL	gene manipulation vector, kan	[26]

pGOAL19	*hyg pAg*_85_*-lacZ sacB Pac*I cassette vector	[26]

pIMP50	pGEM5::*impA*	This study

pIMP51	pGEM5::*impAΔ *(*Sph*I 200 bp)	This study

pIMP57	p2NIL::*impAΔ *(*Sph*I 200 bp)	This study

pFM74	p2NIL::*impAΔ *(769 bp)	This study

pFM75	pFM74 with *Pac*I cassette of pGOAL19	This study

pFM33	p2NIL::*suhB*	This study

pFM48	pFM33::*suhBΔ*	This study

pFM52	pFM48 with *Pac*I cassette of pGOAL19	This study

pFM31	p2NIL::*impC*	This study

pFM53	pFM31::*impCΔ*	This study

pFM54	pFM53 with *Pac*I cassette of pGOAL19	This study

pFM94	pBluescript SK+::*impC *(+288 bp upstream)	This study

pFM96	pFM94::*int gm*	This study

pFM123	pFM96::*impC *D86N	This study

PMN013	plasmid carrying the *M. smegmatis *porin gene *mspA*	[44]

pFM203	pMN013::*int gm*	This study

pFM145	p2NIL::*cysQ*	This study

pFM148	pFM145::*cysQΔ*	This study

pFM151	pFM148 with *Pac*I cassette of pGOAL19	This study

pFM160	pBluescript SK+::*cysQ *(+352 bp upstream)	This study

pFM164	pFM160::*int gm*	This study

### Bioinformatics

Homology searches were carried out using BLASTP ver 2.2.13 [25] The four homologs identified all had e-values <10^-3^, and no other protein match approached significance. Prosite database information was obtained at http://www.expasy.ch/prosite/, using Release 20.56 dated November 4^th^, 2009.

### Construction of *M. tuberculosis *mutants

Targeted mutagenesis was carried out using a two-step strategy [26] in order to introduce an unmarked mutation without any potential polar effects.

### ImpA

Primers tbimpA1 (CTCGACGTACAGGTTGAGCTATCC) and tbimpA2 (CTTCACCTGACCGATCGTCAGCTC) were used to amplify the *impA *gene and flanking regions (2,108 bp) from *M. tuberculosis *H37Rv using PCR. The resulting 2.1 kb fragment was cloned into the *Eco*RV site of pGEM5, producing pIMP50. A 200 bp *Sph*I fragment within *impA *was removed following partial digestion and religated to make pIMP51. The 2,348 bp *Pvu*II fragment of pIMP51 was cloned into p2NIL, producing pIMP57.

To create a deletion where the majority of *impA *was deleted (769 bp deleted from 813 bp), inverse PCR was performed on pIMP57. Primers tbimpAinv1 (TCGTGCCAGCTGACCAACGAATCCAAGTGCAT) and tbimpAinv2 (TCGTGCCAGCTGATAGGGGAACCAGAGGACTA) were used, simultaneously creating a deletion and introducing a *Pvu*II site in the deleted construct. Following the PCR reaction the DNA was digested with *Dpn*I for 1 h at 37C to destroy the template, then digested with *Pvu*II and religated to produce pFM74. Insertion of a *Pac*I gene cassette from pGOAL19 was cloned at the *Pac*I site of pFM74 producing the final delivery plasmid pFM75. The *Pac*I cassette carries *lacZ *and *sacB*, which can be used for positive and negative selection of unmarked mutant colonies, respectively.

### SuhB

A 3,534 bp *Xho*I fragment of cosmid Y5ab was cloned into the *Sal*I site of plasmid p2NIL to produce pFM33. A fragment of 817 bp was deleted from the 874 *suhB *gene by inverse PCR on pFM33 using primers tbsuhBΔ1 (TCAGCATGCGTTCGTTGTCAGGTCGTGTC) & tbsuhBΔ2 (TCAGCATGCGATTCAACGGCCTAGAGC); this introduced a *Sph*I site in the deleted construct. Following treatment with *Dpn*I and *Sph*I, this was religated to produce pFM48. Insertion of the gene delivery cassette from pGOAL19 produced the final delivery plasmid pFM52.

### ImpC

A 2,503 bp *Stu*I fragment of cosmid Y3A2 was cloned into the *Pml*I site of p2NIL, producing pFM31. A 731 bp deletion was generated in the 783 bp gene by inverse PCR on pFM31 using primers tbimpCΔ1 (TGCCAGCTGCATTAGATCGTCGTGGCTCA) & tbimpCΔ2 (TGCCAGCTGGAGGTGCTGACACGGCTC) to introduce a *Pvu*II site in the deleted construct. Following treatment with *Dpn*I and *Pvu*II, this was religated to produce pFM53. Insertion of the delivery gene cassette from pGOAL19 produced the final delivery plasmid pFM54.

### CysQ

Primers tbcysQ1 (CCTGGTCGACCTGTTTCC) and tbcysQ2 (GCGGCTCTTTGACATCTTGT) were used to amplify the *cysQ *gene and flanking regions (2,748 bp) from *M. tuberculosis *H37Rv DNA. The product was cloned into the *Pml*I site of p2NIL, producing pFM145. Primers tbcysQΔ1 (AGTCAGGTCGTCCGTCAGATC) & tbcysQΔ2 (TACAACCAACTGGACCCCTAC) were used to generate a 666 bp deletion in the 804 *cysQ *gene by inverse PCR on pFM145. Following treatment with Klenow polymerase and T4 polynucleotide kinase (Promega), this product was religated to produce pFM148. Insertion of the gene delivery cassette from pGOAL19 produced the final delivery plasmid pFM151.

### Mutagenesis

Deletion plasmids were constructed as described above. The delivery plasmids were introduced into *M. tuberculosis *H37Rv or *M. tuberculosis *H37Rv *ino1*, and single crossovers (SCOs) isolated by selection for blue hyg^R ^kan^R ^colonies. One SCO colony was plated onto 2% (wt/vol) sucrose-50 μg ml^-1 ^X-Gal to isolate bacteria with a second crossover; this will lead to mutant or wild-type cells depending on the location of the recombination event. In order to screen for *impC *mutant, DNA was extracted from sucrose^S ^kan^S ^white colonies (obtained from plating *M. tuberculosis *FAME9 onto sucrose medium) and analysed by PCR using primers that flank the *impC *gene (TBC1: GGACCGCGATCAGTATGAGT and TBC2: TCGACACAGAATCCGCTAGA). Strains carrying the *impC *wild-type allele would produce a band of 1148 bp whereas strains carrying an *impC *mutation would carry the deletion band of 417 bp. Mutant candidates and a wild-type control were digested with *Pvu*II and subjected to Southern blot analysis using a 2.5 kb *impC *probe (*impC *plus flanking region). The wild-type strain showed a 4 kb band whilst the mutant showed a 3.2 kb deletion band along with a 2.5 kb band for the integrated *impC *copy

### Complementation

A construct expressing the *impC *gene was made by PCR amplification of the *impC *gene, together with 288 bp of upstream sequence using chromosomal *M. tuberculosis *H37Rv as template DNA. The primers tbimpCBamP (CGCGGATCCGGCGATGGTGACAT) and tbimpCBam (CGCGGATCCTTACCCGGCGTTGAGC) were used. The product was digested with *Bam*HI and cloned into the *Bam*HI site of pBluescript-SK+ to produce pFM94. The *Hin*dIII cassette of pUC-Gm-int, carrying the *int *and *gm *genes was cloned into the *Hin*dIII site of pFM94 to produce pFM96.

A construct expressing the *cysQ *gene was made by PCR amplification of the *cysQ *gene including 352 bp of upstream sequence using *M. tuberculosis *H37Rv; chromosomal template DNA; primers tbcysup (GCATAGAGCAGGAGGTTTGC) and tbcysend (GCGCCACGCGTCGGCGAT) were used. The PCR product was treated with T4 polynucleotide kinase and cloned into the *Sma*I site of pBluescript-SK+ to produce pFM160. The *Hin*dIII cassette of pUC-Gm-int, carrying the *int *and *gm *genes was cloned into the *Hin*dIII site of pFM160 to produce pFM164.

### Site-directed mutagenesis

Site-directed mutagenesis was carried out using the non-PCR-based Quickchange kit (Stratagene). Oligonucleotides D86N-forward (GGATCGTAGACCCGATCAACGGCACCAAAAACTTTGTGC) & D86N-reverse (GCACAAAGTTTTTGGTGCCGTTGATCGGGTCTACGATCC) were used to prime DNA synthesis with pFM96. Sequencing confirmed the presence of the required mutation.

### Real-time quantitative PCR

RNA was prepared from an exponential (7-day) rolling culture of *M. tuberculosis *H37Rv [27] and cDNA synthesis was carried out using Superscript II (Invitrogen) according to the manufacturer's protocol. Primers were designed for Real-time quantitative PCR (RTq-PCR) for *sigA *(endogenous control), *impA suhB, impC and cysQ) *using the Primer3 software, ensuring products would be less than 500 bp (Table 2). RTq-PCR reactions were set up using the DyNAmo SYBR Green qPCR kit (MJ Research). RT-qPCR was performed using the DNA Engine Opticon 2 System (Genetic Research Instrumentation) using a standard 1 × DNA master SYBR Green I mix, 1 μl cDNA product and 0.3 mM of each primer in 20 μl on ice. The primer concentrations had first been optimised. Samples were heated to 95°C for 10 min before cycling for 35 cycles of 95°C for 30 s, 60°C (*impA, suhB*, *impC *and *cysQ*) or 62°C (*sigA*) for 20 s, and 72°C for 20 s. Fluorescence was captured at the end of each cycle after heating to 80°C to ensure the denaturation of primer dimers. In order to measure relative gene expression levels, standard curves for each primer set were generated by performing PCR with SYBR green detection on serial dilutions of quantified genomic DNA. C_T _values were converted into the equivalent of copy number by comparison to the standard curve. Control reactions where RNA had not been reverse transcribed were used to confirm that there was no significant contaminating genomic DNA present. In order to control for any differences in reverse transcriptase efficiencies each value was standardized to *sigA *to generate unit-less values. *SigA *is a major housekeeping gene and levels of *sigA *mRNA remains constant under a wide range of conditions [28]. Two independent RNA samples were assayed in triplicate for each gene.

**Table 2:** Primers used in Real time quantitative PCR

Gene	Primer pair	Primer sequence
*sigA*	SIGAF	ATCTGCTGGAAGCCAACCT
	SIGAR	GATCACCTCGACCATGTGC

*impA*	IMPAF:	CGATCTCGTCTTCGTCGC
	IMPAR:	CCCTATGCTGCCAAGAATCTC

*suhB*	IMPBF:	GCGAGAAGCAGGCAGAATT
	IMPBR:	CTCTCGGCGTTGACAACAA

*impC*	IMPCF:	GCTGCTTGAAGATGGCGTC
	IMPCR:	CCACCAGGCAGTAAGACAGAA

*cysQ*	CYSQF:	ATCTGACGGACGACCTGACT
	CYSQR:	CCAACGGGTCAATAATCCAC

### Cell wall analysis

#### Extraction and analysis of PIMs

Cells (0.2 g) were delipidated with chloroform/methanol (1/1, v/v) for 48 h at room temperature with continuous stirring. Lipids were separated from the delipidated cells by centrifugation (3000 rpm, 15 min, 2600 *xg*) and analysed by TLC on silica gel-coated plates developed with chloroform/methanol/water (60:35:8, v/v/v). The various PIMs were identified by their mobilities on TLC and their positive reactivity compared to authentic standards; these included a sugar and phospholipid-specific reagent (0.2% anthrone in concentrated H_2_SO_4 _followed by heating) and the Dittmer-Lester reagent that specifically detects phosphorous-containing lipids, respectively [29].

#### Production and analysis of LAM and LM

Delipidated cells were washed and disrupted using a Cell disrupter (2 kbars, Constant System Ltd; one shot model). The resulting material was extracted with 40 mL ethanol/water (1/1, v/v) for 8 h at 65°C; the bacterial residues were discarded and the supernatant was dried. Six ml hot phenol/water (1/1, v/v) were added and the mixture was heated for 1 h at 70°C under continuous stirring, followed by a two-phase partition. The phenol phase was discarded and the upper phase extensively washed and dried. The extract was solubilised in water and Triton X114 (2% wt/v) was added to the cooled suspension. The mixture was stirred for 10 min and then heated at 50°C until two phases formed. The detergent phase was recovered, diluted by adding 1 ml water and washed three times with CHCl3. The resulting aqueous phase was dried to evaporate the chloroform and resuspended in water (0.2 ml). This portion was analysed by SDS-PAGE with a 5% stacking gel and a 15% running gel. Samples were denatured in the presence of 2% SDS in 50 mM Tris-HCl (pH 6.8). After electrophoresis, gels were treated with periodate/ethanol/acetic acid (0.7/40/5, w/v/v), and silver-stained. Authentic samples of mycobacterial LAM and LM from *Mycobacterium bovis *BCG were used as standard.

#### Sugar compositional analysis

The sugar constituents of the various materials were determined after acid hydrolysis with 2 M CF_3_COOH at 110°C for 1 h; the mixture of hydrolysed products was dried, treated with trimethylsilyl reagents [30] to derivatise monosaccharides and analysed by gas chromatography (GC) for their sugars.

#### Gas chromatography and mass spectrometry

GC was performed using a Hewlett Packard HP4890A equipped with a fused silica capillary column (25 m length × 0.22 mm i.d.) containing WCOT OV-1 (0.3 mm film thickness, Spiral). A temperature gradient of 100-290°C at 5°C min-1, followed by a 10-min isotherm plateau at 290°C, was used.

#### Mycothiol assay

Labelling of cell extracts with monobromobimane (mBBr) to determine thiol content was performed with modifications to previously published protocols [31,32]. Cell pellets from 3 ml culture were resuspended in 0.5 ml of warm 50% acetonitrile-water containing 2 mM mBBr (Cal Biochem), and 20 mM HEPES-HCl, pH 8.0. The suspension was incubated for 15 min in a 60°C water bath and then cooled on ice. A final acidic pH was produced by adding 2-5 μl 5 M HCl or 5 M trifluoracetic acid.

The control samples were extracted with 0.5 ml of warm 50% acetonitrile-water containing 5 mM *N*-ethylmalemide and 20 mM HEPES-HCl, pH 8.0. The suspension was incubated for 15 min in a 60°C water bath and then cooled on ice. 2 mM mBBR were added to the solution followed by a second incubation for 15 min in a 60°C. The control sample was cooled but not acidified. Cell debris was pelleted in each sample by centrifugation (5 min 14,000 × g).

HPLC analysis of thiols was carried out by injecting 25 μl of 1:4 dilution of samples in 10 mM HCl on to a Beckman Ultrasphere IP 5 μ(250 mm × 4.6 mm) column using 0.25% glacial acetic acid pH 3.6 (buffer A) and 95% methanol (buffer B). The gradient was: 0 min, 10% B; 15 min, 18% B; 30 min, 27% B; 32 min, 100% B; 34 min, 10% B; and 60 min, 10% B (reinjection). The flow rate was 1 ml min^-1^, and the fluorescence detection was accomplished on a Varian Fluorichrom model 430020 with a 370 nm excitation filter and a 418-700 nm emission filter. Data collection and analysis was performed on Dynamax Mac Integrator (Rainin Instruments).

#### Impase activity

Bacteria were grown to mid-log phase, and collected by centrifugation. Each pellet was washed once in distilled water followed by resuspension in 2.5 ml 2 mM dithiothreitol in 50 mM Tris-Cl, pH8. The suspended bacteria were disrupted in a FastPrep220A at 4 m/sec for 3 cycles of 20 sec in Lysing Matrix B (0.1 mm silica beads), with cooling on ice between cycles. The resulting cell-extracts were then clarified at 4000 g for 4 min using a bench centrifuge and filter-sterilised through 0.2 μm pore cellulose acetate filters (Sartorius Minisart). Each clarified cell extract was desalted through Pharmacia PD-10 columns according to the manufacturer's instructions; with the exception that 3.2 ml (not 3.5 ml) protein fraction was collected. For equilibrating, desalting and eluting using PD-10, 50 mM Tris-Cl, pH8 was used.

Phosphatase assays were conducted using 0.4 mM substrates at 37°C, as described previously [33] although the reaction volume used was 120 μl and was stopped with 30 μl malachite green reagent. No precipitates were formed so the entire assay was performed in ELISA plate wells. Inorganic phosphate present in each well was calculated by reading the OD against a standard curve. Enzyme activity was then calculated by subtracting the phosphate formed in wells with cell extract and substrate, from phosphate formed in corresponding wells with cell extract but without substrate.

## Results

### Bioinformatics analysis

**Figure 1:**
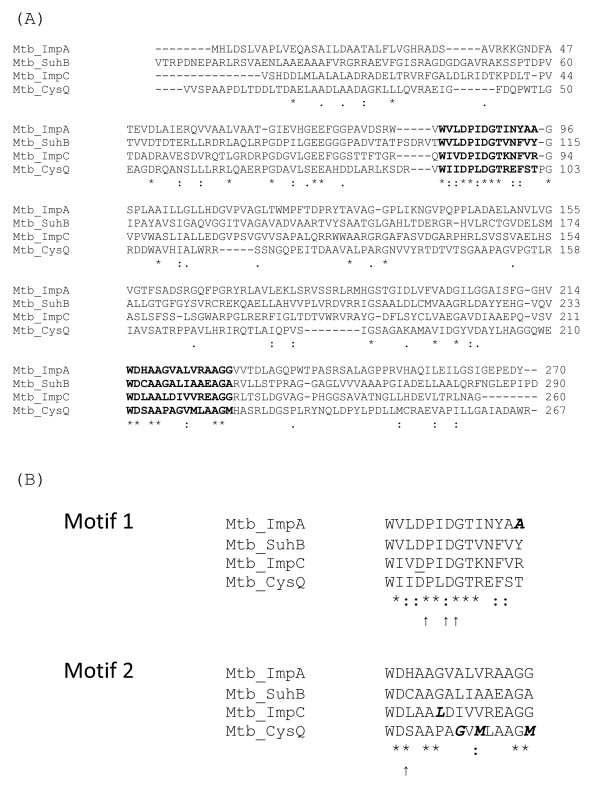
**Alignment of IMPases**. The *M. tuberculosis *H37RvIMPases were aligned using ClustalW. (A) Complete sequences. Motifs shown in bold; (B) Prosite motifs: '*' identical residues in all sequences; ':' conserved substitutions; '.' semi-conserved substitutions. Sequences were obtained from http://genolist.pasteur.fr/TubercuList/. Reported Prosite motifs are 1 (N-terminal; PS00629): [FWV]-x(0,1)- [LIVM]-D-P- [LIVM]-D- [SG]- [ST]-x(2)- [FY]-x- [HKRNSTY]; and 2 (C-terminal; PS00630): [WYV]-D-x- [AC]- [GSA]- [GSAPV]-x- [LIVFACP]- [LIVM]- [LIVAC]-x(3)- [GH]- [GA]. Residues that are not encompassed by these motifs are in bold italics. Arrows indicate putative metal binding aspartate and isoleucine residues reported for human IMPase [55]. The underlined residue shows the aspartate mutated in this study, which is equivalent to mutations introduced into the *E. coli *and human proteins (see main text).

There are four genes in the *M. tuberculosis *genome that encode proteins with significant homology to IMPases. All four *M. tuberculosis *proteins are equally distant from the human IMPase (PDB structure 1IMA; 22-30% identity, 37-46% similarity) [34] and the aligned amino acid sequences are shown in Figure 1A. The four proteins are only as similar to each other, as to the human protein (27-32% identity, 36-44% similarity).

These four genes are generally conserved in other actinomycete genomes, with for example, apparent orthologs in *Mycobacterium avium*, *Mycobacterium smegmatis*, and *Corynebacterium glutamicum *(data not shown). *M. leprae*, which has many pseudogenes, has no functional *impA*. Other genomes do also have a small number of other IMPase genes (thus, *M. avium *has a fifth paralog that is similar to *cysQ*). While levels of homology between the different *M. tuberculosis *IMPase paralogs are moderate (22-30% amino acid identity), similarities between orthologs are much higher (for example, 75-79% identity between *M. tuberculosis *and *M. leprae*, and 51-67% identity between *M. tuberculosis *and *M. smegmatis*).

**Figure 2:**
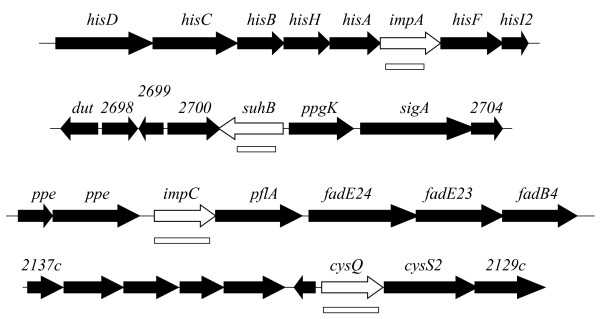
**Genomic context of *M. tuberculosis *IMPase genes**. White arrows: *imp *genes; black arrows: other genes; open rectangles deleted regions in knock out plasmids.

The genomic contexts of these genes are shown in Figure 2. As with *M. smegmatis *[24], the *impA *gene (*Rv1604*) lies in the middle of the main *his *operon between *hisA *and *hisF*. The stop codon of *hisA *overlaps with the putative start codon of *impA*, and the stop codon of *impA *overlaps with the putative start codon of *hisF*. These *impA *genes are 70% identical.

The *suhB *gene (*Rv2701c*) was named in the original genome annotation [35], because it is the gene most similar to the *Escherichia coli suhB *gene. The *E. coli suhB *gene was so-named because deletion of the gene resulted in a cold-sensitive phenotype, and suppression of a thermosensitive *rpoH *mutation [36]. It has also been shown to suppress *secY *[37], *dnaB *[38], and *era *[39] mutations. However, these phenotypes are not related to the enzymatic properties of the protein, as they are unaffected by a null point mutation in the active site [40] (Figure 1B). Furthermore, inositol production is not believed to occur in *E. coli*, so the biological context is very different from that in mycobacteria. Recombinant SuhB from *M. tuberculosis *has been confirmed to have IMPase activity [41]. *SuhB *is monocistronic in *M. tuberculosis *(Figure 2).

The third homologous gene is *Rv3137*, which we have called *impC*. It appears to be the first gene in a two-gene operon; a 457 bp intergenic gap upstream of *impC *suggests it has its own promoter., and a second gene, *pflA*, is predicted to start only 14 bp downstream, so is probably co-transcribed. PflA shows homology to pyruvate formate lyase-activating proteins. Beyond this is a cluster of *fad *genes (*fadE24-fadE23-fadB4*), but the gap beyond *pflA *and *fadE24 *is 79 bp, so is less likely to be part of the same operon.

The fourth homologous gene is *cysQ *(*Rv2131c*), so-named because it is most similar to the *E. coli cysQ *gene. *E. coli cysQ *mutants are cysteine auxotrophs during aerobic growth [42]. Interestingly *M. smegmatis *contains two paralogs of this gene.

Two sequence motifs have been described for IMPases in the Prosite database [43] (see legend to Figure 1B). One motif, near the N-terminus contains the metal-binding aspartate residues of the active site, and the other lies near the C-terminus. All of the gene products except SuhB had small differences from at least one of the two IMPase motifs (Figure 1B). However, they all contain the important metal-binding residues in both motifs.

### The *M. tuberculosis impA *and *suhB *genes are dispensable

The *impA *gene has previously been shown to play an indirect role in inositol synthesis in *M. smegmatis *[24], and a knockout plasmid construct was therefore prepared to isolate an *M. tuberculosis impA *mutant. As the gene lies within the *his *operon (Figure 2), this plasmid carried an unmarked deletion that would not have polar effects. The mutant was generated using a two-step method [26], and grew well on solid medium. Unlike the *M. smegmatis impA *mutant which had altered colony morphology, there were no obvious differences in colony morphology between the wild-type and mutant strains.

We carried out a similar experiment to determine whether *suhB *plays a role in inositol metabolism. Again, a deletion construct was prepared, and an unmarked mutant isolated, with no obvious differences in colony morphology.

### Inactivation of CysQ

We constructed a plasmid to delete the *cysQ *gene. Initially, we were unable to obtain a mutant; of 97 double crossovers (DCOs) screened in the presence of inositol, all were wild-type. We therefore made a merodiploid strain by integrating a second copy of *cysQ *into the single crossover (SCO) strain, and repeating the selection for DCOs on sucrose. Using this method, 24 out of 30 colonies were found to be mutants. The ability to isolate a mutant only in the presence of a functional copy of the gene indicates that this gene was essential under the conditions tested. It could be inferred that *cysQ *synthesizes all the inositol in the cell, or all the inositol for a specific essential molecule. However, this hypothesis is improbable, as, if true, we would predict thatmutants would be inositol auxotrophs, yet no mutants were isolated even in the presence of high levels of inositol. One possibility is that inositol does not penetrate the cell wall, which is known to be highly impermeable. However, as we had successfully isolated a mutant lacking inositol-1-phosphate synthase (an inositol auxotroph), only when the media was supplemented with extremely high levels of exogenous inositol (50-77 mM) [23], it seems that inositol does enter the cell in sufficient quantities but permeability to this molecule is poor. This suggests that even a slight increase in the requirement for inositol might make mutant isolation impossible, since we had reached the limits of inositol solubility. We reasoned that an increase in the availability of inositol by introduction of a porin might allow a mutant to be isolated. We therefore electroporated an integrating plasmid (pMN013) carrying the *M. smegmatis *porin gene *mspA *[44,45] (for which *M. tuberculosis *has no orthologue) into the SCO strains, and repeated the sucrose selection. Using this method, we successfully isolated a *cysQ *mutant in the presence of 77 mM inositol. We screened 16 DCO colonies and two were mutants.

We then plated the mutant on inositol-free medium, and were surprised to observe normal growth, indicating that once the mutant has been isolated, it does not require inositol. To determine whether the mutant could survive in the absence of the *mspA *gene, the integrating plasmid containing this gene was switched with an empty integrative plasmid carrying the hygromycin resistance gene (pUC-Hyg-int). This would result in the replacement of the *cysQ*-carrying plasmid, leaving a stain with no functional *cysQ*. Surprisingly, we were able to obtain *cysQ *mutants using this approach although we had failed to isolate a mutant by our standard mutagenesis procedure. We therefore conclude that *cysQ *is also dispensible, and a *cysQ *mutant does not require inositol for growth.

### The *impC *gene is essential

We attempted to construct an unmarked *impC *deletion mutant. The first step of the mutagenesis to produce SCOs worked well, however, when cells carrying a second crossover were isolated, only wild-type bacteria were obtained. In theory, the second crossover could take place on either side of the deletion, resulting in either mutant or wild-type cells. The fact that we obtained only wild-type cells (n = 48) suggested that the mutants are not viable.

These initial mutagenesis experiments were carried out in the absence of exogenous inositol. We therefore repeated the mutagenesis, including different levels of inositol in the media at all times. Again, only wild-type bacteria were isolated following the second cross-over (n= 97; 16 on 15 mM inositol, 8 on 30 mM, 16 on 46 mMl, and 57 on 77 mM).

The inability to obtain a mutant may be due to other factors, such as a low frequency of recombination on one side of the gene, even though the length of flanking DNA should be sufficient (847 and 874 bp). Therefore we constructed a merodiploid strain by inserting a second functional copy of *impC *into the SCO strain. This extra copy was present on an L5-based integrating vector, and contained 288 bp upstream of *impC*, which was likely to carry its promoter. When this strain (FAME9) was plated onto sucrose to isolate DCOs, three out of eight colonies isolated had lost the original copy of *impC*. The fact that this gene could only be lost when a second copy of the gene is present suggests that *impC *is essential for survival, even in the presence of high levels of exogenous inositol (Fisher's exact test, p < 0.01, comparing only the experiments with 77 mM inositol and the complemented strain). To further investigate the essentiality of the *impC *gene, and in view of what was observed with *cysQ*, we introduced the *mspA *gene into the *impC *SCO strain; this time we were not successful in obtaining a mutant, indicating that the difficulty we encountered making an *impC *mutant differed from *cysQ*.

A difference between an IMPase mutant and an *ino1 *mutant may be that inositol-1-phosphate accumulates in the IMPase mutant, which might somehow be detrimental to the cell. We therefore carried out the essentiality experiment in an *ino1 *mutant background. The *impC *mutant construct was introduced into *M. tuberculosis ino1*, and a SCO strain isolated. On plating for DCOs, only wild-type colonies were isolated (n = 38), again suggesting that this was not the explanation for the essentiality.

### Site directed mutagenesis of *impC*

Our results suggest that *impC *does not have a critical role in inositol production and hence our inability to obtain an *impC *mutant may indicate that *impC *has a different or secondary function that prevents isolation of a mutant. For example, the enzyme might form part of an enzyme complex, and play a vital structural role in maintaining the integrity of that complex. Deletion of the gene would then have both enzymatic and structural effects. An analogous situation was found with the *E. coli *SuhB protein; where phenotypes in *suhB *mutants were not related to IMPase activity, as a point mutation in the active site did not produce the suppressing phenotype [40]. We therefore used the same approach to try to separate enzymatic activity from a structural role.

A D93N change in *E. coli SuhB *and an equivalent D90N change in the human IMPase suppress activity [40,46] (Figure 1B). Site-directed mutagenesis was used to introduce a corresponding mutation (D86N) in the *M. tuberculosis impC *gene using the integrating plasmid pFM96 previously used for complementation. This plasmid (pFM123) was introduced into the SCO strain FAME7, and the resultant strain (FAME11) was streaked onto sucrose/inositol plates. DCO colonies were analysed, and, in contrast to the situation with pFM96, all were shown to be wild-type (n = 52). The fact that the functional *impC *gene could not be replaced by this mutated gene, even in the presence of inositol (p < 0.01), shows that the mutation did inactivate enzymatic activity, and (assuming that the structure was not affected) that it is this enzymatic activity that is essential, rather than an additional structural role.

### Enzyme activities

In order to gain a greater understanding of the function of these IMPases, we expressed *impC *as a recombinant protein. However, despite using different plasmid constructs and strategies, we were unable to obtain a soluble protein (not shown). As an alternative to directly assaying enzyme activity, we assayed IMPase activity in cell extracts of the mutant strains to obtain information about their relative contributions to inositol synthesis. We compared enzyme activities in whole cell extracts from the wild-type and mutant strains (Tables 3 and 4). Of the seven substrates tested, phosphate release as a result of adding the enzyme source was significantly higher than controls for fructose bisphosphate (FBP), the inositol phosphates, 5' AMP and *p*-nitrophenyl-phosphate. Deletion of the *impA*, *suhB*, or *cysQ *genes made no significant difference to IMPase activity. The *cysQ *mutants had significantly less FBPase than the parent strain, (P < 0.05; t-test). However, the fructose FBPase activity in the H37Rv control for the *cysQ *mutants (Table 4) is significantly less than in H37Rv control used for *impA *and *suhB *mutants (P < 0.05; t-test) (Table 3) suggesting that the small but significant differences reported in this study may be due to batch-to-batch variation rather than in relation to any mutations.

**Table 3:** Phosphatases in cell extracts of *impA, suhB *mutants

Substrate	H37Rv	Δ**impA**	Δ**suhB**
Fructose-1,6-bisP	26.04 ± 1.85 (5)	28.18 ± 0.92 (5)	32.70 ± 0.44 (5)

Inositol-1-P	0.63 ± 0.13 (6)	0.79 ± 0.12 (5)	0.63 ± 0.25 (6)

Inositol-2-P	1.20 ± 0.15 (4)	1.33 ± 0.22 (5)	1.03 ± 0.15 (6)

Glycerol-2-P	0.08 ± 0.06 (12)	-0.02 ± 0.03 (2)	0.39 ± 0.03 (2)

Glycerol-3-P	-0.13 ± 0.12 (12)	-0.08 ± 0.03 (2)	0 ± 0.21 (2)

5' AMP	4.22 ± 0.36 (8)	4.13 ± 0.40 (2)	5.74 ± 0.04 (2)

*p*-nitrophenyl-P	3.00 ± 0.35 (12)	3.55 ± 0.14 (2)	4.38 ± 0.36 (2)

Values: nmol/min/mg protein, mean ± SEM (n). Differences between levels in mutants and the parent strain were not significant (P > 0.05; t-test).

**Table 4:** Phosphatases in cell extracts of the *cysQ *mutants

Substrate	H37Rv	Δ***cysQ *203/12**	Δ***cysQ*203/16**
Fructose-1,6-bisP	18.94 ± 1.00 (6)	13.09 ± 1.24 (6)	12.41 ± 0.54 (7)

Inositol-1-P	0.40 ± 0.09 (8)	0.49 ± 0.17 (9)	0.57 ± 0.16 (9)

Inositol-2-P	0.84 ± 0.12 (8)	0.90 ± 0.27 (10)	0.70 ± 0.23 (10)

Glycerol-2-P	0.75 ± 0.32 (8)	1.02 ± 0.27 (10)	0.55 ± 0.15 (10)

Glycerol-3-P	-0.37 ± 0.28 (3)	-0.35 ± 0.14 (3)	0.27 ± 0.45 (3)

5' AMP	1.42 ± 0.31 (3)	1.69 ± 0.14 (3)	1.39 ± 0.03 (3)

*p*-nitrophenyl-P	5.51 ± 0.36 (2)	3.64 ± 1.92 (2)	2.83 ± 0.25 (3)

Values: nmol/min/mg protein, mean ± SEM (n). Level of FBPase in *cysQ *mutants relative to parent strain is significantly different (P < 0.05; t-test). Level of FBPase in H37Rv parent strain reported in table 4 is significantly different (P < 0.05; t-test) to that reported in Table 3.

### PIM, LAM and mycothiol levels are normal in the *impA*, *suhB *and *cysQ *mutants

**Figure 3:**
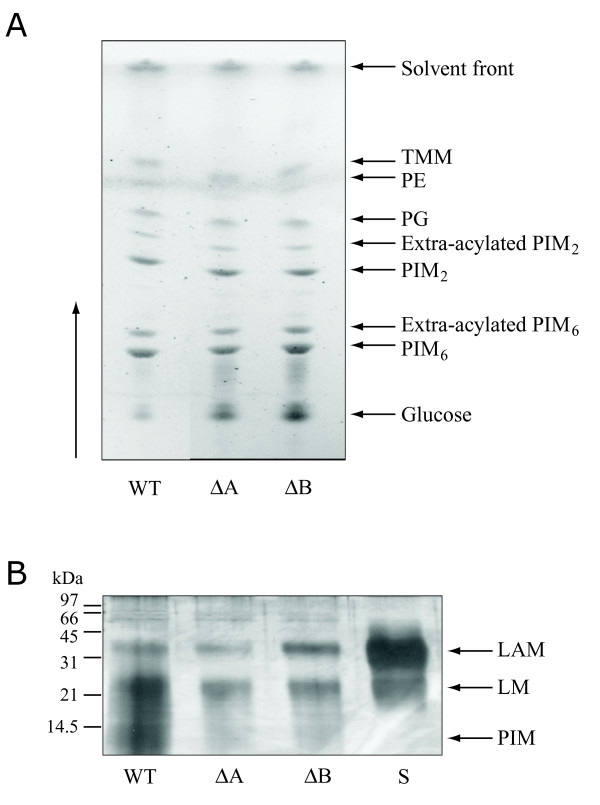
**Analyses of cell wall major constituents of some representative mutants**; the other strains exhibited profiles similar to those shown. (A) TLC analysis of extractable lipids. (B) SDS-PAGE of lipopolysaccharides. WT: *M. tuberculosis *H37Rv; ΔA: *impA *mutant; ΔB: *suhB *mutant; S: authentic standard of mycobacterial LAM and *M. bovis *BCG LM; TMM: trehalose monomycolate; PE: phosphatidylglycerol; PG: phosphatidylethanolamine; LAM: lipoarabinomannan; LM: lipomannan; PIM: phosphatidylinositol mannoside.

**Figure 4:**
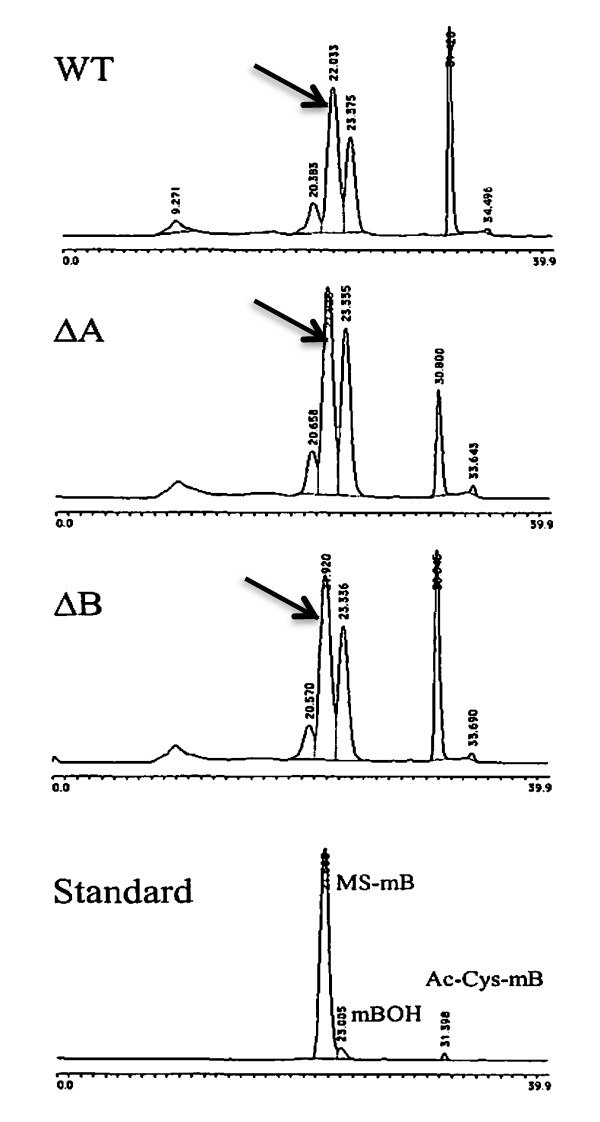
**HPLC analysis of mycothiol (marked with an arrow) in representative mutants**; the other strains exhibited profiles similar to those shown. WT: *M. tuberculosis *H37Rv; ΔA: *impA *mutant; ΔB: *suhB *mutant. Free thiol peaks are marked as standard (lower panel) MS-mB marks the mycothiol peak and and Ac-Cys-mb represent an Acetyl-Cysteine thiol.

Cell extracts of the mutant strains were prepared for the assay of inositol-containing molecules (cell envelope glycolipids and mycothiol). TLC analyses showed that PIMs were normal in the mutant strains (Figure 3A), whilst polyacrylamide gel electrophoresis (Figure 3B) and sugar compositional analysis (not shown) demonstrated normal levels of LAM and LM. Mycothiol levels were assayed by HPLC analysis; levels in the *impA*, *suhB and cysQ *mutants were similar to wild-type (see Figure 4).

### Gene expression levels of imp genes in *M. tuberculosis*

The relative contributions of the IMPase homologues will be proportional to their activity, and their level of expression. We therefore carried out RTq-PCR experiments to determine the levels of expression of *impA, suhB, cysQ and impC *mRNA in exponential cultures of *M. tuberculosis*. Expression levels were normalized to those of *sigA *mRNA which remains constant. The level of *cysQ *was the highest, almost equal to *sigA *(Table 5). *impA *and *impC *were expressed at approximately 40% of this level, while *suhB *was lowest, at 12% of the *cysQ *level.

**Table 5:** mRNA levels

Gene	mRNA level normalised to sigA*
***impA***	0.41 (0.3- 0.5)

***suhB***	0.11 (0.096- 0.13)

***impC***	0.36 (0.27- 0.46)

***cysQ***	0.95 (0.76- 1.18)

*To ensure equal amounts of cDNA were used each value was standardized to *sig A *to generate unit-less values

(95% confidence interval)

## Discussion

To investigate how *M. tuberculosis *synthesises inositol, we carried out a genetic analysis of four IMPase homologues in *M. tuberculosis*. The *impA *and *suhB *genes were shown to be dispensable, with no phenotype detected in terms of the levels of mycothiol, PIMs, LM or LAM. *CysQ *is also dispensible, although isolating the mutant proved more difficult, requiring introduction of the *M. smegmatis mspA *porin gene for mutant isolation, but not for subsequent survival. It cannot be excluded, however, that the *cysQ *mutants that were eventually obtained had acquired a suppressor mutation, which had allowed deletion of *cysQ *or had allowed growth of the mutant on media lacking inositol and preventing cell death. In contrast to these three genes, we were only able to inactivate *impC *by introducing a second copy of the gene. The TraSH mutagenesis protocol which provides a genome-wide indication of essentiality [47] supports our data, with only *impC *of these four genes being reported as putatively required for optimal growth *in vitro*.

Inositol production is likely to be essential for mycobacterial growth, because of the essentiality of both classes of mycobacterial inositol-containing molecules, namely phospholipids [8] and mycothiol Our previous work showing that a PI synthase mutant is an inositol auxotroph [23] is consistent with this. Both SuhB and CysQ have been shown to have IMPase activity [41,48] and we have shown that the *M. smegmatis *ImpA has IMPase activity (unpublished data). However, none of the three mutants constructed are auxotrophic for inositol, indicating that there is potential redundancy of function between the homologs and deletion of three or four genes might be required to see sufficient loss of activity to cause auxotrophy. A recent report suggests that CysQ is likely to play a role in sulphur metabolism, as its activity as 3'-phosphoadenosine-5'-phosphatase is several orders of magnitude higher than as an inositol phosphatase [49]. However, it may still contribute to the redundancy in inositol phosphatase activity.

To determine the potential relative contribution of each of these genes to inositol synthesis we carried out two experiments. One experiment looked at the relative amounts of mRNA using real-time RTq-PCR. All mRNA species were detectable, with *cysQ *being most abundant (approximately the same level as *sigA*, the major housekeeping sigma factor), and *impA *being the least abundant, with a level only one-tenth that of *cysQ*. We also assayed the level of IMPase activity in the whole cell extracts of each mutant, reasoning that we might see a decrease in activity when one of the genes was deleted. However, no decrease in activity was observed in any of the three mutants compared to the wild-type strain. This could be a reflection on the sensitivity of our assay, or could indicate that the activity is regulated (either at the transcriptional or post-transcriptional level) such that a constant level is maintained.

We also have preliminary data that expression of the *impC *gene is regulatable. We grew a Δ*ino1 *mutant of *M. tuberculosis *(which needs >50 mM inositol for its normal growth [23]) and looked at the effect of removal of the inositol on gene expression. The only IMPase gene with changed expression was *impC*, which was 3-fold increased. We cannot link this change directly to the inositol, because it could also be caused by the change in osmolarity, but at the very least indicates this indicates this gene is regulatable (unpublished results).

The situation with *impC *is complicated in that we could neither obtain a mutant, nor do we have biochemical evidence that it functions as an IMPase (despite many attempts to achieve both). The essentiality cannot be a simple case of *impC *producing the majority of the inositol in the cell, as we added inositol exogenously. It is true that the *ino1 *mutant we made previously, which is an inositol auxotroph, required levels of inositol approaching the maximum solubility limit, so a requirement for a slightly increased level of inositol might explain our findings. However, this is unlikely because (i) we also introduced a porin gene to increase inositol uptake, with no effect, (ii) we would also have to explain why the other three IMPase genes are not sufficient, and (iii) the level of *impC *mRNA is only 21% of the total IMPase mRNA (41% if *cysQ *is excluded). The only pieces of evidence we have, therefore, that link *impC *to inositol production are (i) its clear homology to IMPases, and (ii) the circumstantial evidence that levels of *impC *increased in a microarray experiment where inositol was removed from an *ino1 *auxotroph, whereas the expression level of the other IMPase genes was not significantly changed. We recognise the difficulty of carrying out the latter experiment in a controlled way since removing such a high level of inositol from the medium could have other effects. Interestingly, *impC *was also upregulated in the Wayne low oxygen model, particularly when *M. tuberculosis *cultures entered a microaerophilic state known as nonreplicating persistent stage 1 (NRP1), where there is cessation of bacterial replication, strong induction of respiratory nitrate reductase activity, and a change in energy metabolism (3.3-fold induced) [50].

We tested the hypothesis that the essentiality of *impC *is unrelated to its enzymatic activity by constructing a site-directed mutation. The mutation introduced changes at an active-site of glutamate to glutamine; the analogous mutation has been shown to abrogate activity in the human protein [40,46]. Our inability to isolate mutants, strongly suggests that (i) the point mutation does indeed affect the activity of the enzyme and (ii) *impC *carrying this point mutation cannot complement a null mutant even in the presence of inositol. These findings oppose our hypothesis of a structural role for ImpC, and support an enzymatic role, as an explanation of its essentiality. There still remains a possibility that the mutation also affects the structure as we have not shown that folded protein is still produced, but we believe this is unlikely given the subtle nature of the change introduced. Another possible explanation for the inositol-independent essentiality is that removal of ImpC results in a build up of inositol-1-phosphate, which is somehow deleterious to the cell. However, we were unable to obtain an *impC *mutant in an *ino1 *background. It is feasible that ImpC uses a substrate other than inositol i.e. one involved in mycothiol production. The elegant work of Fahey and co-workers has defined most of the mycothiol biosynthesis pathway, but is missing a predicted phosphatase., which dephosphorylates N-acetyl glucosamine-(α1,3)-1L-inositol-1-phosphate. We carried out preliminary experiments attempting to make an *impC *mutant using this substrate (kindly provided by R. Fahey and G. Newton), without success (not shown). However, we have no evidence that it would penetrate the cell, so we feel we cannot draw any conclusions.

The *impC *gene lies upstream of the *pflA *gene and may be co-transcribed, as the intergenic gap is only 19 bp. *PflA *shows homology to pyruvate formate lyase-activating proteins; oxygen-sensitive iron-sulfur proteins that activate an anaerobic ribonucleotide reductase in some bacteria [51], although there does not appear to be a homologue to *E. coli *pyruvate formate lyase in the *M. tuberculosis *genome. We designed an unmarked deletion of *impC*, in order to prevent polar effects. In addition, complementation with *impC *alone was sufficient to allow mutants to be isolated. We have therefore excluded polar effects on *pflA *as an explanation for the essentiality.

The *Mycobacterium leprae *genome contains many pseudogenes therefore genomic comparisons may give an indication as to which mycobacterial genes are essential. In *M. leprae*, the *impA *orthologous gene is a pseudogene, with several frameshifts in the distal half of the gene, whereas the other three orthologous IMPase genes are retained. The *suhB *orthologous gene (*ML1024*) appears to be functional, and has a similar genomic context to the *M. tuberculosis *gene. The orthologous *impC *gene (*ML0662*) appears to be monocistronic in this species, and the orthologous *cysQ gene *(*ML1301*) is also present. The lack of phenotype in an *M. tuberculosis impA *mutant contrasts with the situation seen in *M. smegmatis*, where an *impA *mutant had altered colony morphology, slower growth, and reduced levels of PIM_2 _[24]. The fact that the *M. smegmatis *mutant is viable supports the idea of some redundancy of function, and we suggest that the differences in phenotype are caused by different levels of ImpA compared to other IMPases in the two species.

Given that inositol monophosphatase and fructose-bisphosphatase activities were detected in cell extracts from *impA*, *suhB *and *cysQ *mutants, none of these genes can encode the major enzyme for these activities. The *cysQ *gene product does in fact act as a phosphatase with fructose-1,6- bisphosphate and inositol-1-phosphate [48], but enzyme activity in assays does not always equate to functionality in living bacteria. An example is found in *Thermococcus kodakarensis *where knocking out the *fbp *gene encoding a fructose bisphosphatase with high substrate specificity resulted in a strain unable to grow on gluconeogenic substrates whilst knocking out its *imp *gene encoding a member of the carbohydrate phosphate superfamily with substrate specificity including fructose-1,6- bisphosphate did not affect its growth on any carbon sources [52]. In *M. tuberculosis*, the effect of knocking out the *glpX *gene that encodes fructose bisphosphatase is so drastic it is difficult to envisage that *impA, suhB *or *cysQ *can compensate for its loss [53].

## Conclusions

We have demonstrated that the *M. tuberculosis impA*, *suhB *and *cysQ *genes are dispensable, but that *impC *is essential under the growth conditions used. The reason for the essentiality is unclear in terms of inositol synthesis; at present the most attractive hypothesis is that *impC *is required for mycothiol synthesis.

## Authors' contributions

FM carried out the molecular genetic studies, participated in the design and coordination of the study and drafted the manuscript. PW conceived of the study, carried out the enzyme assays and wrote the corresponding section of the manuscript. PD performed cell wall analysis. MD designed the cell wall analysis and aided in drafting the manuscript. YA conceived of the study, designed and carried out the Mycothiol assay. TP conceived of the study, participated in the design and coordination, and aided in drafting the manuscript. NS conceived of the study, participated in its design and coordination, performed the bioinformatics and participated in drafting the manuscript. All authors read and approved the final manuscript.
